# Supplementary material for: Identification, Expression and Evolutional Analysis of Two cyp19-like Genes in Amphioxus
Source: Animals (Basel). 2024 Apr 9;14(8):1140. doi: 10.3390/ani14081140 (PMC11047327; doi:10.3390/ani14081140)
Supplement: Supplementary file 1 [file animals-14-01140-s001.zip › animals-2918434-supplementary.pdf]

Table S1. The GenBank accession numbers .

**Title 1**
NP_000094.2 aromatase [*Homo sapiens*]
NP_001335100.1 aromatase [*Mus musculus*]
XP_046780576.1 aromatase [*Gallus gallus*]
NP_001090630.1 CYP19A1 [*Xenopus tropicalis*]
XP_043533302.1 aromatase [*Chiloscyllium plagiosum*]
XP_048418626.1 aromatase [*Stegostoma fasciatum*]
XP_020386362.2 aromatase [*Rhincodon typus*]
XP_038669275.1 aromatase [*Scyliorhinus canicula*]
XP_041030878.1 aromatase [*Carcharodon carcharias*]
XP_032906075.1 aromatase [*Amblyraja radiata*]
XP_042199999.1 aromatase [*Callorhinchus milii*]
XP_038850963.1 aromatase-like [*Salvelinus namaycush*]
XP_014030724.1 aromatase [*Salmo salar*]
XP_026865672.2 aromatase [*Electrophorus electricus*]
XP_026877222.2 brain aromatase [*Electrophorus electricus*]
XP_042608630.1 brain aromatase [*Cyprinus carpio*]
XP_042599607.1 ovarian aromatase-like [*Cyprinus carpio*]
XP_026031446.1 brain aromatase-like [*Astatotilapia calliptera*]
XP_026019473.1 aromatase [*Astatotilapia calliptera*]
XP_005450866.1 brain aromatase [*Oreochromis niloticus*]
NP_571229.3 aromatase [*Danio rerio*]
XP_021326198.1 CYP19A1b [*Danio rerio*]
NP_001265808.1 aromatase [*Oryzias latipes*]
XP_011474159.1 CYP19A1b [*Oryzias latipes*]
XP_035672840.1 aromatase-like [*Branchiostoma floridae*]
XP_035669280.1 aromatase-like [*Branchiostoma floridae*]
XP_019626033.1 aromatase-like [*Branchiostoma belcheri*]
XP_019639573.1 aromatase-like [*Branchiostoma belcheri*]
XP_032801472.1 aromatase [*Petromyzon marinus*]
XP_033105836.1 aromatase-like [*Anneissia japonica*]
XP_057290659.1 aromatase-like [*Hydractinia symbiolongicarpus*]
NP_001189784.1 cytochrome P450 3A4 isoform 2 [*Homo sapiens*]
BAB20498.1 cytochrome P450, CYP3A [*Mus musculus*]
NP_001001751.2 cytochrome P450 family 3 subfamily A member 5 [*Gallus gallus*]
NP_997838.1 cytochrome P450, family 3, subfamily c, polypeptide 1 [*Danio rerio*]
NP_001292334.1 cytochrome P450, family 3, subfamily A [*Oryzias latipes*]
AGO32790.1 Cyp3A [*Azumapecten farreri*]
CAB4069313.1 CYP3A [*Lepeophtheirus salmonis*]
CAG2198897.1 CYP3A [*Mytilus edulis*]
CAE1256603.1 CYP3A [*Sepia pharaonis*]
AAA52140.1 CYP17A [*Homo sapiens*]
AAT01928.1 CYP17A [*Mus musculus*]
AUD11671.1 steroid 17-alpha-hydroxylase/17,20 lyase [*Anser cygnoides*]
AAT01120.1 cytochrome P450c17 [*Chelydra serpentina*]
NP_001090540.2 cytochrome P450 family 17 subfamily A member 1 [*Xenopus laevis*]
AAP41821.1 ovarian cytochrome P450c17 [*Danio rerio*]
AAR88432.1 P450c17 [*Anguilla japonica*]
NP_001118219.1 steroid 17-alpha-hydroxylase/17,20 lyase [*Oncorhynchus mykiss*]

WLF94733.1 steroid 17-alpha-hydroxylase/17,20 -like protein [*Apostichopus japonicus*]
NP_001187242.1 steroid 17-alpha-hydroxylase/17,20 [*Ictalurus punctatus*]
NP_001266694.1 steroid 17-alpha-hydroxylase/17,20 -like [*Oreochromis niloticus*]
XP_020555398.1 steroid 17-alpha-hydroxylase/17,20 isoform X1 [*Oryzias latipes*]
BAF61104.1 cytochrome P450 17alpha-hydroxylase/17, 20- [*Branchiostoma belcheri*]
AAA52140.1 CYP17A [*Homo sapiens*]
AAT01928.1 CYP17A [*Mus musculus*]
AUD11671.1 steroid 17-alpha-hydroxylase/17,20 lyase [*Anser cygnoides*]
AAT01120.1 cytochrome P450c17 [*Chelydra serpentina*]
NP_001090540.2 cytochrome P450 family 17 subfamily A member 1 L [*Xenopus laevis*]
AAP41821.1 ovarian cytochrome P450c17 [*Danio rerio*]
AAR88432.1 P450c17 [*Anguilla japonica*]
NP_001118219.1 steroid 17-alpha-hydroxylase/17,20 lyase [*Oncorhynchus mykiss*]
WLF94733.1 steroid 17-alpha-hydroxylase/17,20-like protein [*Apostichopus japonicus*]
NP_001187242.1 steroid 17-alpha-hydroxylase/17,20 lyase [*Ictalurus punctatus*]
NP_001266694.1 steroid 17-alpha-hydroxylase/17,20 lyase-like [*Oreochromis niloticus*]
XP_020555398.1 steroid 17-alpha-hydroxylase/17,20 lyase isoform X1 [*Oryzias latipes*]
AAB59440.1 steroid 21-hydroxylase [*Homo sapiens*]
BAA31153.1 steroid 21-hydroxylase [*Mus musculus*]
AFN53625.1 CYP21 [*Chrysolophus pictus*]
BAF63009.1 steroid 21-hydroxylase [*Gallus gallus*]
NP_001087443.1 cytochrome P450 family 21 subfamily A member 2, 1 L [*Xenopus laevis*]
BAC76051.1 cytochrome P450 21-hydroxylase [*Anguilla japonica*]
KAB7505058.1 Steroid 21-hydroxylase [*Armadillidium nasatum*]
